# Parallel In Vitro and In Silico Studies of the Anti-Inflammatory Activity of Bioactive Compounds Found in Different Ethanolic Extracts of Bracts from *B. x buttiana* (var. Rose): A Comparative Analysis

**DOI:** 10.3390/ph18060821

**Published:** 2025-05-30

**Authors:** Gabriela Castañeda-Corral, Mayra Cedillo-Cortezano, Vera L. Petricevich

**Affiliations:** Facultad de Medicina, Universidad Autónoma del Estado de Morelos (UAEM), Calle Leñeros, Esquina Iztaccíhuatl S/N. Col. Volcanes, Cuernavaca C.P. 62350, Morelos, Mexico; gabriela.castaneda@uaem.mx (G.C.-C.); mayra.cedillo@docentes.uaem.edu.mx (M.C.-C.)

**Keywords:** *B. x buttiana*, ethanol concentration, in vitro anti-inflammatory activity, phospholipase A2, cyclooxygenase, protein denaturation, erythrocyte membrane stabilization, in silico analysis

## Abstract

**Background/Objectives:***Bougainvillea x buttiana* is used in traditional Mexican medicine to treat various diseases. Previous studies have demonstrated its anti-inflammatory properties, which are associated with its chemical composition. This study evaluated the effect of ethanol concentration on the yield and anti-inflammatory activity of its extracts. Additionally, an in silico analysis of the plant’s previously identified phytochemicals was conducted. **Methods:** Four extracts of *B. x buttiana* (var. Rose) (labeled as BxbREE) were prepared with increasing concentrations of ethanol (0%, 50%, 80%, and 100%). Their anti-inflammatory activity was assessed using different in vitro assays. The in silico prediction, performed with SwissADME, included the physicochemical, pharmacokinetic, and drug-like properties of the compounds. **Results:** The findings indicated that varying the ethanol concentration in the preparations of BxbREE-0%, BxbREE-50%, BxbREE-80%, and BxbREE-100% significantly impacted the extraction yield, with BxbREE-0% and BxbREE-50% exhibiting the highest recovery. All four extracts demonstrated significant anti-inflammatory activity, with BxbREE-50% and BxbREE-80% showing the most important effects on the denaturation of bovine serum albumin (BSA) and trypsin, inhibition of pro-inflammatory enzymes (cyclooxygenase and phospholipase A2), and increased stability of the erythrocyte membrane. The in silico analysis revealed that most phytochemicals identified in the extracts had good drug-likeness and bioavailability for oral administration and an adequate ADME profile. **Conclusions:** These findings reaffirm the anti-inflammatory potential of *B. x buttiana* (var. Rose) ethanolic extracts and the favorable pharmacokinetic and pharmacodynamic properties of its phytochemicals. Further structural exploration of the interactions of these bioactive compounds could contribute to the design of new drugs.

## 1. Introduction

Inflammation is a natural defense mechanism that helps fight infections, burns, and harmful stimuli and prepares the body for healing [1]. Inflammation increases blood flow, enhances the permeability of blood vessels, and causes protein denaturation. This denaturation leads to the formation of type III hypersensitivity-associated antigens, which might worsen inflammation and trigger autoimmune diseases. Protein denaturation occurs due to external factors such as heat, organic solvents, and strong acids, all of which can alter the secondary and/or tertiary structures of proteins. When enzymes lose their structural integrity, substrates cannot bind to the active site, resulting in activity loss [2,3].

Inflammation is progressively known as a contributing factor to the development of various diseases, leading to growing requirements for anti-inflammatory medications [4]. Nonsteroidal anti-inflammatory drugs (NSAIDs) like ibuprofen, diclofenac, and indomethacin are commonly prescribed for their effectiveness in reducing pain and inflammation. These effects are due to the inhibition of cyclooxygenase (COX), resulting in reduced prostaglandin synthesis and the prevention of protein denaturation [5,6]. However, the use of NSAIDs is associated with several side effects, including gastrointestinal toxicity and risks of cardiovascular diseases, as well as liver and kidney damage [7]. Consequently, there is growing interest in natural compounds with anti-inflammatory properties.

Many species in the plant kingdom are known for their medicinal properties, which have been used historically in traditional medicine to treat inflammatory conditions [8]. Scientists have identified secondary metabolites with therapeutic properties through extensive chemical, pharmacological, and toxicological studies of plant extracts [9]. Today, many approved medications are made from these bioactive plant metabolites [10]. From a chemical point of view, secondary metabolites are classified as alkaloids (which include hormones and essential oils), phenolic compounds (comprised of coumarins, flavonoids, lignin, and tannins), glycosides (consisting of saponins), and terpenes/terpenoids [9].

Research showed that phenolic compounds in the diet have important anti-inflammatory and antioxidant properties. Accordingly, consuming foods rich in these compounds may lower the risk of chronic diseases, such as cardiovascular diseases and cancer, suggesting a need for further study on their phytochemical and pharmacological effects [11,12]. The polyphenol family includes over 8000 molecules widely distributed throughout the plant kingdom. Polyphenols have one or more aromatic rings along with one or more hydroxyl moieties in their chemical structure Their antioxidant properties are influenced by the number of hydroxyl groups present and the distance between the carbonyl group and the aromatic ring [11,13]. The extraction of compounds with antioxidant properties from plants requires the use of appropriate extraction methods and solvents, as well as proper temperature control. Evidence suggests that polar solvents, such as aqueous mixtures containing ethanol, are the most effective for enhancing the extraction of polyphenol [14].

Bougainvillea is frequently utilized in many countries, including Mexico, to treat numerous diseases [15]. Antecedently, we reported that extracts from the pink and orange bracts of *B. x buttiana* displayed significant antioxidant and anti-inflammatory properties both in vitro and in vivo, likely due to the presence of distinct secondary metabolites in the extracts [12,16,17].

In the midst of this, the total phenolic content extracted with ethanol from the bracts of *B. x buttiana* was critical for the observed antioxidant effect [12,16,18,19]. However, some studies showed that plant extracts obtained with different extraction methods exhibit significant differences in biological activity, highlighting the importance of selecting appropriate extraction techniques to optimize the yield of bioactive compounds. This study planned to intensify our comprehension of how solvent concentration influences the composition of *B. x buttiana* (var. Rose) extracts and their in vitro anti-inflammatory actions. To further confirm these activities, we analyzed and compared them by the following methods: (a) inhibition of trypsin and bovine serum albumin (BSA) denaturation; (b) inhibition of the pro-inflammatory enzymes cyclooxygenase (COX) and phospholipase A2 (PLA2); and (c) stabilization of the erythrocyte membrane. Additionally, an in silico study was conducted to evaluate the physicochemical and ADME properties of the metabolites previously identified in the extracts to determine which ones exhibit the most promising drug-like properties.

## 2. Results

### 2.1. Comparison of the Amount of Ethanol and the Yield

To estimate the effect of ethanol content on the yield, composition, and activity of extracts elaborated from *B. x buttiana* bracts, four extracts were prepared using the following *v/v* ethanol quantities: -0%, -50%, -80%, and -100%. Three independent extractions were performed for each extract. The extracts were identified as BxbREE-0%, BxbREE-50%, BxbREE-80%, and BxbREE-100%. The weights of the dried extracts were used to calculate the yield. The recovery percentages of BxbREE-0% and BxbREE-50% were 30.31% and 29.28%, respectively. In descending order, the extract yields were BxbREE-0% = BxbREE-50% > BxbREE-80% > BxbREE-100%. However, it was observed that increasing the quantity of ethanol significantly minimized the recovery percentages of BxbREE-80% and BxbREE-100% (Table 1).

### 2.2. Ethanol Concentration’s Impact on In Vitro Anti-Inflammatory Action

#### 2.2.1. Impact of the BxbREE on Protein Denaturation

**Figure 1:**
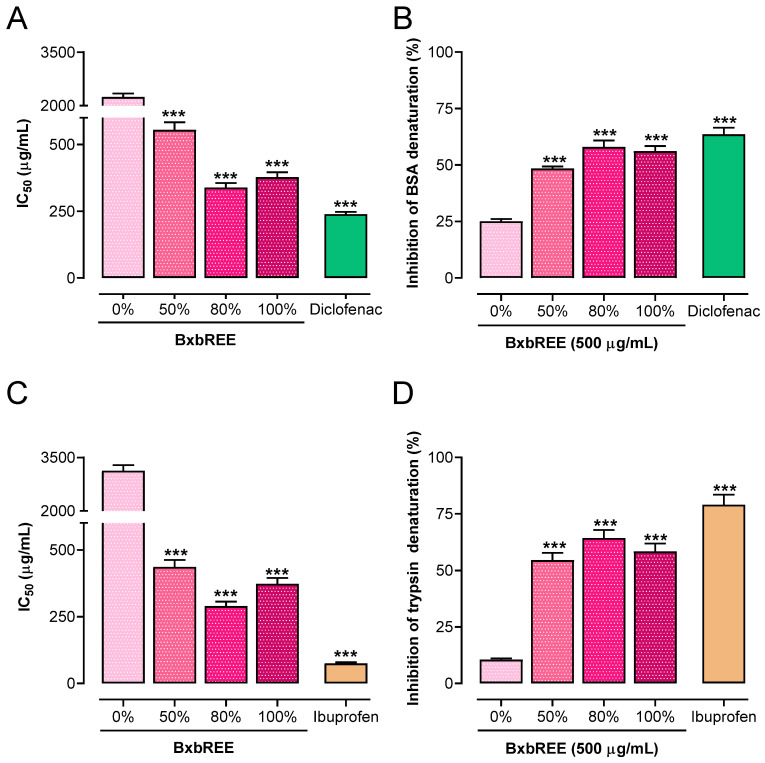
Effects of BxbREE-0%, BxbREE-50%, BxbREE-80%, and BxbREE-100% on the inhibition of BSA or trypsin denaturation. (**A**,**C**): Representation of the calculated half inhibitory concentration (IC_50_) values of each BxbREE. (**B**,**D**): Inhibition percentage of BSA or trypsin denaturation of each extract at 500 μg/mL. Data are expressed as the mean from three independent extractions wherein each extraction was evaluated (*n* = 4) ± the standard error of mean. The symbol described mentions significant differences *** (*p* < 0.001) vs. BxBREE-0% by one-way ANOVA followed by the Dunnett test.

Two methods, heat-induced BSA denaturation and trypsin denaturation assays, were used to estimate the impact of the ethanolic extracts of *B. x buttiana* on inhibiting protein denaturation. The findings showed that each BxbREE was able to inhibit BSA and trypsin denaturation within a concentration range of 100 to 500 µg/mL. For the heat-induced BSA denaturation assay, the calculated IC_50_ values were as follows: 554.64 µg/mL, 338.42 µg/mL, and 377.3 µg/mL for BxbREE-50%, BxbREE-80%, and BxbREE-100%, respectively. Notably, all IC_50_ values for the extracts were significantly higher than those from the diclofenac-treated groups (238.82 µg/mL; *p* < 0.001; Figure 1A). The highest percentages of BSA denaturation inhibition were observed at a concentration of 500 µg/mL for all extracts. The inhibition percentages, in descending order, were diclofenac (63.59%), BxbREE-80% (58.0%), BxbREE-100% (56.1%), BxbREE-50% (48.4%), and BxbREE-0% (25.1%) (Figure 1B). Similar results were observed regarding the percentages of trypsin denaturation inhibition. The calculated IC_50_ values for BxbREE-50%, -80%, and -100% were 436.56 µg/mL, 289.44 µg/mL, and 373.05 µg/mL, respectively (Figure 1C). These IC_50_ values were at least eight times lower than the IC_50_ for BxbREE-0% (3126.3 µg/mL), but significantly higher than the IC_50_ for ibuprofen (74.7 µg/mL) (*p* < 0.001). The percentages of inhibition of trypsin denaturation by BxbREE with ethanol were 10.5%, 54.6%, 64.3%, and 58.4% for BxbREE-0%, -50%, -80%, and -100% (Figure 1D). In BxbREE-80%, the inhibition percentages were slightly higher when compared to the other extracts. These results suggest that increasing the ethanol concentration in the extract obtention enhances the inhibitory percentage of BSA and trypsin denaturation compared to BxbREE-0%.

#### 2.2.2. Effect of BxbREEs on Phospholipase Activity

**Figure 2:**
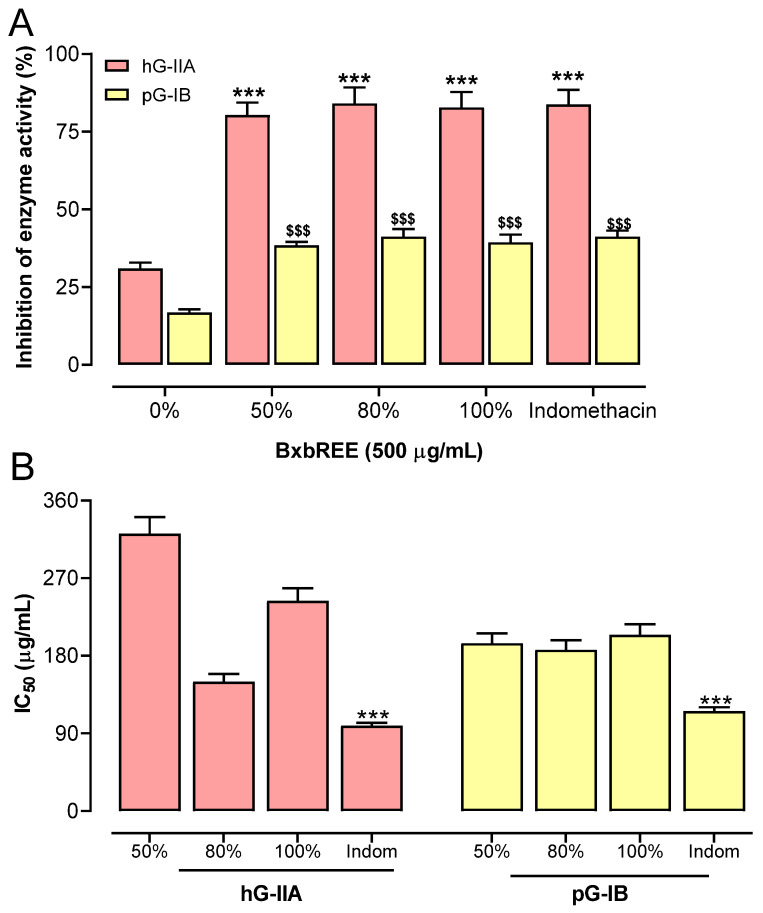
BxbREE-0%, -50%, -80%, and -100% significantly inhibited the activity of phospholipase A2. Panel (**A**). Effect of BxbREE on the activity of phospholipase A2 (pro-inflammatory group IIA and group IB digestive) at 500 μg/mL. Panel (**B**). Estimated values of 50% inhibitory concentration (IC_50_). Data are expressed as the mean from three independent extractions when each extraction was evaluated as (*n* = 4) ± the standard deviation. The different symbols above, *** and $$$, correspond to *p* < 0.001 vs. the BxBREE-0% group by one-way ANOVA followed by the Dunnett test.

The anti-inflammatory activities of BxbREE-0%, -50%, -80%, and -100% were evaluated in vitro at different concentrations. The ability of the extracts to selectively inhibit pro-inflammatory group IIA phospholipase A2 compared to digestive group IB phospholipase A2 was determined. The secreted phospholipases hG-IIA and pG-IB were used in the assays. All extracts studied were found to inhibit hG-IIA and pG-IB. The effect of each extract at 500 µg/mL on hG-IIA and pG-IB activity is shown in Figure 2A. BxbREE-0% was found to inhibit hG-IIA activity by 31%. Additionally, the highest inhibitions of hG-IIA activity were from BxbREE-50% (80.4%), BxbREE-50% (84.1%), and BxbREE-100% (82.8%). Among these extracts, the highest percentage of inhibition was obtained in BxbREE-80%. In addition, the inhibition percentages of pG-IB activity in BxbREE-0% were an average of 16.8%, and in BxbREE-50%, -80%, and -100% were 40% (Figure 2A). The inhibitions of hG-IIA (83.8%) and pG-IB (41.2%) were obtained with indomethacin treatment. The values of IC_50_ for the groups of hG-IIA and pG-IB are illustrated in Figure 2B. It was observed that the IC_50_ values of hG-IIA groups were found within a range of 149.97 µg/mL and 321.52 µg/mL, and for indomethacin treatment, 98.90 µg/mL. It is important to emphasize that BxbREE-80% showed the lowest IC_50_ value. Towards the IC_50_ values of pG-IB, we found that they were similar and ranged between 186.95 µg/mL and 204.35 µg/mL for the extracts, while for indomethacin, this was 115.80 µg/mL (Figure 2B).

#### 2.2.3. BxbREEs’ Effects on Cyclooxygenase Activity

**Figure 3:**
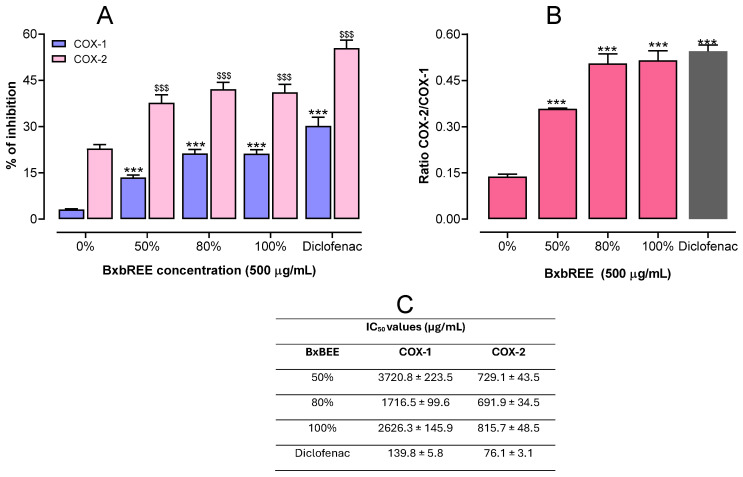
Effects of BxbREEs on the inhibition of COX-1 and COX-2. (**A**). Inhibitory activity of the extracts at a concentration of 500 µg/mL. (**B**). COX-1/COX-2 ratio. (**C**). Calculated 50% inhibitory concentration (IC_50_) values for each sample extract. Data are expressed from three independent extractions where each extraction was evaluated as the mean (*n* = 4) ± standard deviation. The different symbols described, *** and $$$, reveal significant differences (*p* < 0.001) vs. the BxBREE-0% group by one-way ANOVA followed by the Dunnett test.

The effects of BxbREEs extracted at different ethanol concentrations on the activities of cyclooxygenase-1 (COX-1) and cyclooxygenase-2 (COX-2) enzymes were tested. The results showed that the different BxbREEs at a concentration of 500 µg/mL were able to significantly inhibit both COX-1 and COX-2 activities. For COX-1 activity, the percentages of inhibition were 3.17% to 21.3%. However, for COX-2 activity, the inhibition percentages ranged from 22.83 to 42.1%, best observed in BxbREE-80%. These results suggest that all BxbREEs were more efficient in inhibiting COX-2 when compared to COX-1 (*p* < 0.001) (see Figure 3A). The percentages of inhibition obtained for diclofenac were 30.20% and 55.40% for COX-1 and COX-2, respectively. Similarly, the inhibition ratios of COX-1 to COX-2 were 0.138 for BxbREE-0%, 0.358 for BxbREE-50%, 0.505 for BxbREE-80%, and 0.515 for BxbREE-100%, and for diclofenac was 0.545, which confirms that BxbREEs had greater selectivity for COX-2 (Figure 3B). These findings are reinforced by the estimated values of IC_50_, which were significantly higher for COX-1 than for COX-2 (see Figure 3C). It is worth mentioning that the increase in ethanol concentration for the obtention of the extracts did not alter their effects on COX-1 and COX-2 activities. Between BxbREE-50% and BxbREE-100%, the inhibition percentages were similar. Meanwhile, in BxbREE-80%, a slightly increased inhibition percentage was observed.

### 2.3. BxbREEs’ Effects on Erythrocyte Membrane Integrity Assay

**Figure 4:**
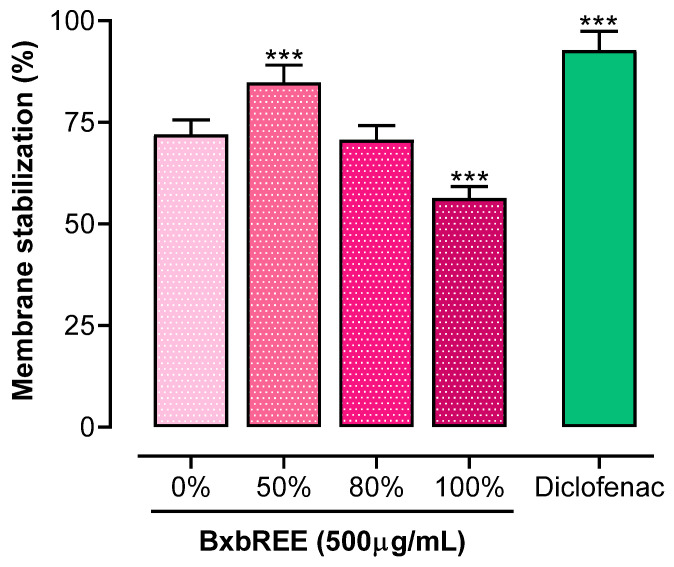
BxbREEs’ effects on the erythrocyte membrane. Data are expressed from three independent extractions and each extraction was evaluated as the mean (*n* = 4) ± standard deviation of mean values (*n* = 3). The symbols *** indicate significant differences (*p* < 0.001) vs. the BxBREE-0% group by one-way ANOVA followed by the Dunnett test.

The effects of the four BxbREEs at a concentration of 500 µg/mL on erythrocyte membrane stabilization are shown in Figure 4. The results indicated that all extracts significantly inhibited hemolysis, with a percentage of membrane stabilization ranging from 56.35 to 84.88%. However, the efficacy of these extracts was lower in comparison with the reference drug diclofenac, which achieved a stabilization percentage of 92.74%. Among the extracts, BxbREE-50% exhibited the highest effectiveness (84.88%) in stabilizing erythrocyte membranes, while BxbREE-0% and BxbREE-100% showed statistically equal stabilization percentages of 72 and 70.68%, respectively. In contrast, BxbREE-100% had the lowest effect of inhibiting hemolysis, with a stabilization percentage of 56.35%. These findings suggest that increasing the ethanol concentration to 80% to prepare the extracts did not substantially alter their stabilizing activity.

### 2.4. In Silico Analysis of the Compounds Found in BxbREE-0%, -50%, -80%, and -100%

Modern drug development is expensive and time-consuming. It evaluates the efficacy and potency of molecules, and their ability to reach biological targets in cells, animals, and humans. In silico tools are a faster alternative that can forecast the pharmacokinetic, physicochemical, and medicinal properties of small molecules, aiding in optimizing drugs for disease treatment [20,21].

In previous studies, we reported the chemical composition of *B. x buttiana* (var. Rose) extracts obtained using solvents such as acetone, ethanol, and methanol. However, to date, it is not yet clear which metabolite or groups of metabolites may be responsible for the antioxidant and anti-inflammatory activities. Specifically, sixteen compounds were identified in the ethanolic extracts, suggesting that these compounds may be partially responsible for the reported antioxidant and anti-inflammatory effects [12,16]. Based on the above, to identify the metabolites with the most promising pharmacological properties, we performed an in silico study using SwissADME, a free web tool, accessed in October and November 2024. The molecular descriptors predicted for the compounds present in the different BxbREEs were the Lipinski rule of five parameters (R05), log water solubility (*Log S*), bioavailability score, and synthesis accessibility score [21,22]. The 16 listed compounds have molecular weights below 500 Daltons, fewer than five hydrogen bond donors (HBDs), and less than six hydrogen bond acceptors (HBAs). Eight of the sixteen compounds exhibited a Log *P* value of less than five. In summary, all the compounds meet the criteria of Lipinski’s rule of five. Water solubility (*Log S*) is essential for absorption after oral administration, since a drug must be in an aqueous solution to be absorbed. The results showed that the solubility profile of the compounds was as follows: **C-1** to **C-6** and **C-10** are soluble to highly soluble in water, **C-7** to **C-9**, **C-11**, **C-12**, **C-14**, and **C-15** have moderate to poor solubility, while **C16** shows low solubility. Notably, 15 of the 16 BxbREE compounds have a synthetic accessibility score between 1 and 3.79, indicating that they can be moderately or easily synthesized for further studies (Supplementary Table S1). These results together suggest that the bioactive compounds present in BxbREE have suitable physicochemical properties and a high probability of being absorbed orally.

**Figure 5:**
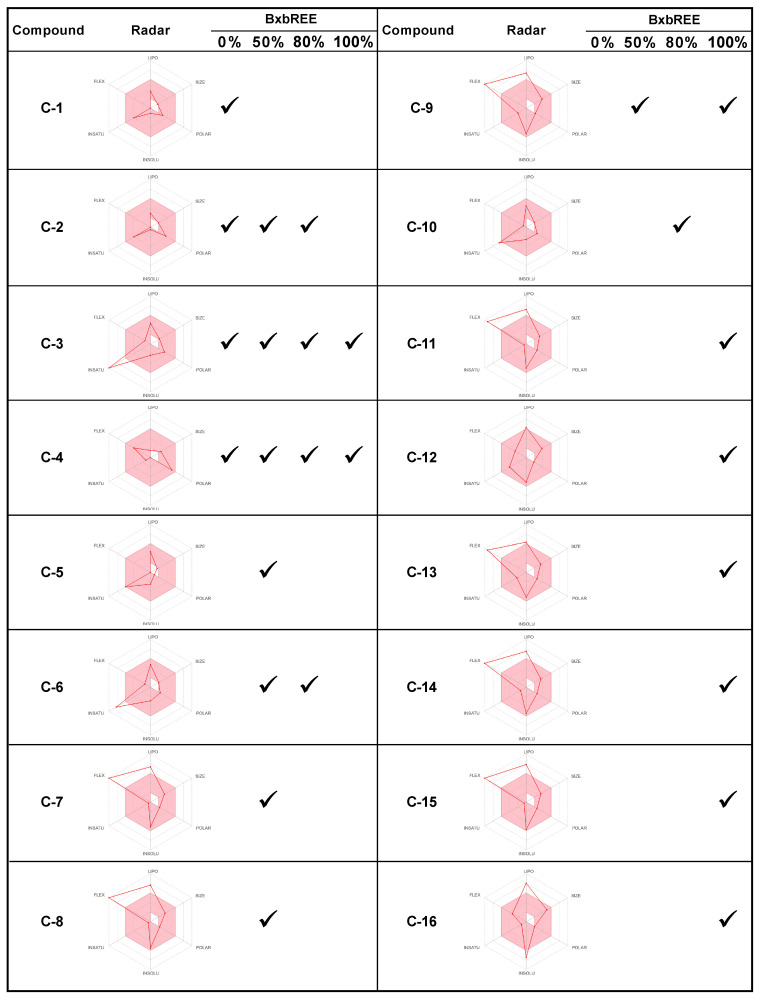
Bioavailability radar plots of the compounds identified in BxbREE-0%, -50%, -80%, and -100%. Liposolubility (LIPO), molecular weight (SIZE), polarity (POLAR), insolubility (INSOLUB), insaturation (INSATU), and flexibility (FLEX). The pink area represents the optimal range for each property (lipophilicity: XLOGP3 between −0.7 and +5.0; size: MW between 150 and 500 g/mol; polarity: TPSA between 20 and 130 Å 2; solubility: Log S not higher than six; saturation: fraction of carbons in the sp 3 hybridization not less than 0.25; and flexibility: no more than nine rotatable bonds [21]).

Drug-likeness evaluates the likelihood that an unknown molecule will have a bioavailability similar to known drugs, making it suitable for oral administration. This concept is a complex balance of several molecular and structural properties, including hydrophobicity (LIPO), electronic distribution (INSATU), *Log S* not higher than six (INSOLUB), polarity (POLAR), molecular weight (SIZE), and flexibility (FLEX) [21]. To be classified as drug-like, a molecule must completely fall within the designated pink area of the radar plot. To conduct an initial assessment of drug-likeness, we created a bioavailability radar plot for each secondary metabolite identified in the four BxbREEs. We also provide the number and types of metabolites identified in each extract (Figure 5). The bioavailability radar plots showed that compounds **C-1, C-2, C-4, C-5, C-10**, and **C-12** fell in the pink, suggesting they exhibit good drug-like properties. In contrast, compounds **C-3** and **C-6** were slightly above the ideal unsaturation, while compounds **C-7–C-9**, **C-11**, and **C-13–C-16** exhibited slightly higher than desirable lipid solubility and flexibility. BxbREE-0% contained four metabolites (**C-1** to **C-4**); BxbREE-50% had eight metabolites (**C-2** to **C-9**); BxbREE-80% had five compounds (**C-2**–**C-4**, **C-6**, and **C-10**); and BxbREE-100% contained nine metabolites (**C-3**, **C-4**, **C-9**, and **C-11** to **C-16**). BxbREE-50% and BxbREE-100% were the extracts with the highest number of compounds. However, BxbREE-50% and BxbREE-80% were found to contain compounds with the most favorable drug-like properties, which may explain their highest anti-inflammatory activity in vitro. In contrast, while BxbREE-100% contains the largest number of compounds, it was found to be the least effective. This could be due, in part, to the fact that most of the compounds in this extract exhibit low to moderate solubility, high flexibility, and high lipid solubility.

**Figure 6:**
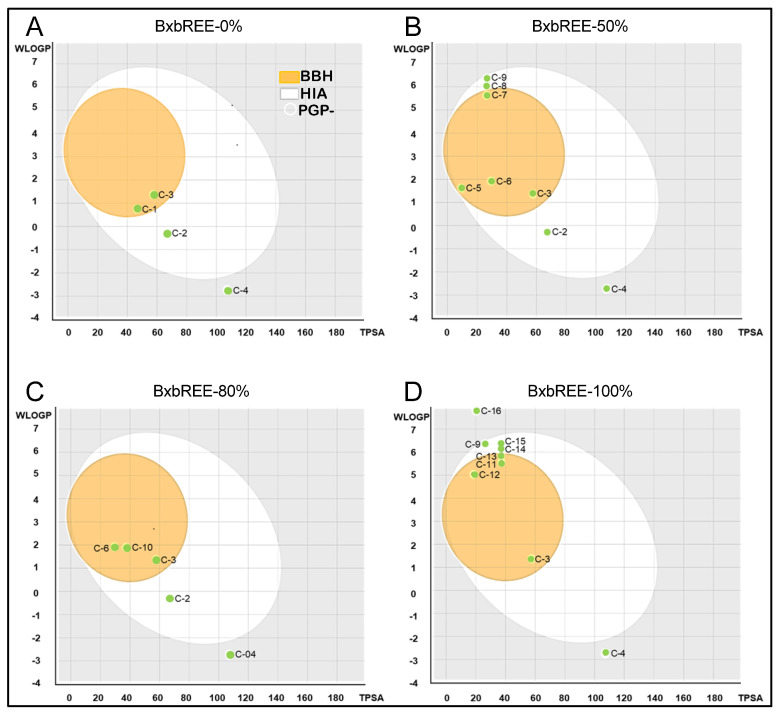
BOILED-Egg model of phytochemicals present in BxbREE-0% (**A**), BxbREE-50% (**B**), BxbREE-80% (**C**), and BxbREE-100% (**D**). The white region represents a high probability of human passive gastrointestinal absorption (HIA), and the yellow region represents a high probability of penetration through the BBB. The green color indicates that the compound is not actively effluxed by P-glycoprotein (PGP).

In the present study, the predictions of human gastrointestinal absorption (HIA), blood–brain barrier (BBB) penetration, and P-glycoprotein (P-gp)-mediated efflux of each compound were plotted in the BOILED-Egg model [20]. For BxbREE-0%, three out of four compounds exhibited high HIA, and compounds **C-1** and **C-3** showed a high probability of BBB penetration (Figure 6A). In the case of BxbREE-50%, seven out of eight compounds demonstrated high HIA, while **C-3**, **C-5–C-7** have a high probability of BBB penetration (Figure 6B). For BxbREE-80%, four out of five compounds showed high human intestinal absorption (HIA), and only three compounds (**C-3**, **C-6**, and **C-10**) had a high probability of BBB penetration (Figure 6C). In BxbREE-100%, seven out of nine compounds exhibited high HIA, with only **C-3** and **C-11** to **C-13** demonstrating high BBB penetration (Figure 6D). However, compounds **C-4** and **C-16** did not meet these parameters. In summary, of the 16 secondary metabolites identified in the ethanolic extracts of *B. x buttiana*, 14 exhibited high HIA, and eleven demonstrated high BBB penetration. P-gp is an ATP-dependent drug transporter that plays a key role in intestinal absorption, metabolism, and transport across the blood–brain barrier. It also transports hydrophobic compounds out of the cells, contributing to drug resistance [23]. Therefore, it is important to determine whether a bioactive compound is a P-gp substrate or inhibitor. We found that the 16 compounds were neither substrates nor inhibitors of P-gp (Figure 6). Altogether, these results suggest that most of the bioactive compounds studied presented high HIA, good bioavailability, and can efficiently cross the BBB without being affected by P-gp-mediated efflux.

Metabolism is a key process that prevents xenobiotics, including drugs, from reaching toxic concentrations through enzymatic reactions. The cytochrome (CYP) P450 superfamily is involved in phase I of the oxidative metabolism of drugs [24]. This study identified five phenolic compounds (**C-3, C-5, C-6, C-10**, and **C-12**) among the phytochemicals of *B. x buttiana*. **C-3** (2-propenoic acid, 3-(2-hydroxyphenyl)-, (E)-) was found in BxbREE-0%, -50%, -80%, and -100%; C-5 (Benzofuran, 2,3-dihydro-) was found in BxbREE-50%; **C-6** (2-Methoxy-4-vinylphenol) was present in BxbREE-50% and -80%; **C-10** (Ethanone, 1-(2-hydroxy-5-methylphenyl)-) in BxbREE-80%; and **C-12** (Naphthalene, 3,4-dihydro-1,8-bis(trimethylsilyloxy)- in BxbREE-100%.

Due to its significant antioxidant and drug-like properties, we evaluated the probability of these molecules to be substrates or inhibitors of the most important CYP450 enzymes (Table 2). The analysis performed using SwissADME revealed that all five phenolic compounds were neither substrates nor inhibitors of CYP450 2C9, CYP450 2D6, nor CYP450 3A4, except for **C-12**, which was predicted as a CYP450 3A4 substrate. **C-3** and **C-6** were not found to be inhibitors, while **C-5** and **C-12** were identified as inhibitors of CYP450 1A2 and CYP450 2C19. Lastly, compound **C-10** was predicted to be an inhibitor of CYP450 1A2 but not of CYP450 2C19. Overall, these results showed that four of the five phenolic compounds displayed low CYP inhibitory promiscuity, except for **C-12**. These results suggest that phenolic compounds from *B. x buttiana* have a low risk of drug interactions.

**Figure 7:**
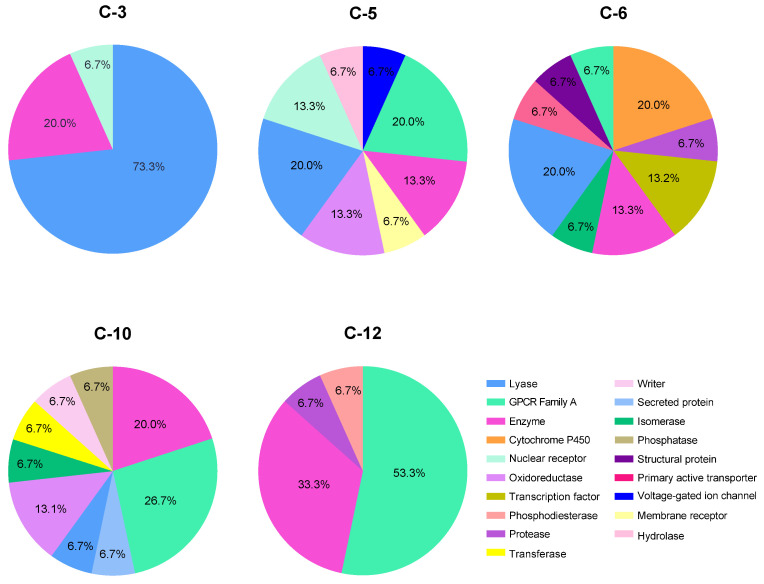
Prediction of the top 15 human molecular targets for the phenolic compounds identified in the different BxbREEs.

To better understand the pharmacodynamics of *B. x buttiana* secondary metabolites, particularly phenolic compounds, we performed ligand-based target prediction using the SwissTargetPrediction tool (accessed October and November 2024) [25]. Our analysis showed that lyases, enzymes, and the G protein-coupled receptor (GPCR) family were the most likely drug targets for phenolic compounds. With these targets, **C-1** and **C-12** exhibited an interaction probability of 93.3% and 92.3%, respectively. **C-5**, **C-6**, and **C-10** showed interaction probabilities of 53.3%, 40%, and 53.7%, respectively. Additionally, these compounds could interact with at least six other classes of drug targets, including proteases, CYP450 enzymes, nuclear receptors, and transcription factors (Figure 7).

## 3. Discussion

The plant extraction process is crucial for obtaining pharmacologically active secondary metabolites [12]. To extract these compounds efficiently, the polarity of the solvent and the physicochemical properties of the plant constituents must be considered. Commonly used organic solvents for medicinal extracts include methanol, pure ethanol (EtOH), and ethanol–water mixtures [26,27].

This study analyzed the effect of EtOH concentration on the preparation, yield, and in vitro anti-inflammatory activity of *B. x buttiana* bract extracts. Four extracts, named BxbREE-0%, BxbREE-50%, BxbREE-80%, and BxbREE-100%, were prepared using the following ethanol/water mixtures as solvents: 0%, 50%, 80%, and 100%. The results showed that increasing the EtOH concentration significantly reduced recovery. This suggests that water influences yield, as aqueous extracts contain various compounds, including carbohydrates, that are highly soluble and can impact the overall yield [26,27].

Inflammation, particularly chronic inflammation, plays a significant role in the development and progression of many diseases [4]. Nowadays, many plant extracts have been widely studied for their potential in the treatment of inflammatory conditions [28]. In agreement, several types of plant-derived secondary metabolites, such as phenols, terpenoids, flavonoids, saponins, and tannins, have demonstrated anti-inflammatory activity [27,29].

Based on ethical considerations, this study used the following well-validated in vitro assays to compare the anti-inflammatory activity of BxbREEs: BSA and trypsin denaturation inhibition assays, PLA2, COX-1, and COX-2 enzyme activity inhibition, and the erythrocyte membrane integrity assay. Protein denaturation is an important factor in inflammatory diseases such as arthritis and diabetes [30]. Reducing protein denaturation may help control these inflammatory conditions. In this study, all four BxbREEs at 500 µg/mL significantly inhibited heat-induced BSA and proteinase denaturation. BxbREE-50%, -80%, and -100% produced the highest percentages of denaturation inhibition, with no significant differences observed between these extracts. In contrast, BxbREE-0% displayed the lowest protein denaturation (25.1% and 10.5%) for BSA and trypsin, respectively. This result suggests that increasing the EtOH concentration enhanced the inhibitory denaturation activity.

Eicosanoids such as leukotrienes, thromboxane, and prostaglandins are pro-inflammatory mediators derived from arachidonic acid through the action of the enzymes phospholipase A2 and cyclooxygenase. Decreasing eicosanoid synthesis by inhibiting PLA2, COX-1, and COX-2 activity reduces inflammation. Our results showed that all extracts were competent to inhibit the enzymatic activity of PLA2 extracellular or secreted subgroups (PLA2G-IB and PLA2G-IIA), COX-1, and COX-2. All extracts selectively inhibited the activity of the PLA2 hG-IIA subgroup. BxbREE-0% inhibited approximately 31%, while BxbREE-50%, -80%, and -100% inhibited approximately 82%. Furthermore, BxbREE-50%, -80%, and -100% also effectively inhibited COX-1 and, with greater potency, COX-2. These results are consistent with a previous study performed in vivo, which showed that an acetone extract and ethanolic extracts of *B. x buttiana* inhibited these three pro-inflammatory enzymes and prevented protein denaturation in the treatment of arthritis, respectively [17,31].

Stabilizing the lysosomal membrane prevents inflammation during tissue damage by avoiding the release of lytic enzymes [32]. The erythrocyte membrane stabilization method is a rapid tool that resembles the lysosomal membrane stabilization effect, which means that bioactive compounds affecting erythrocyte membranes may also impact lysosomal membranes, thus preventing inflammation [33,34]. In this study, the membrane stabilization percentages differed for BxbREE-0%, -50% (84.88%), -80% (84.88%), and -100% (56.35%), while the stabilization percentage of the reference drug diclofenac was 92.74%. This result suggests that the concentration of ethanol used to make the extract significantly affects the stabilization membrane effect of the extracts.

There is much evidence that demonstrates that the mechanisms relating to the anti-inflammatory effects of natural compounds can be evaluated by investigating membrane stabilization. In this direction, the inhibition of the enzyme that metabolizes arachidonic acids (PLA2 and PG synthase), reduced the release of inflammatory mediators, generation of NO, and mobilization of inflammatory cells. Thus, the PLA2 inhibition by extracts can be evaluated as its ability to stabilize the membrane, preventing the release of free phospholipids from the erythrocyte membrane or even by direct inhibition of the release or action of PLA2. Overall, the results of this study show that the ethanolic extracts of *B. x buttiana* displayed significant anti-inflammatory activity by inhibiting protein denaturation and the activity of pro-inflammatory enzymes, stabilizing the erythrocyte membrane and preventing the release of harmful enzymes and other inflammatory substances. The in vitro anti-inflammatory action added to the results previously obtained from in vivo tests could be assigned to the presence of bioactive compounds in BxbREE. These include flavonoids, glycoside derivatives, alkanes, esters, terpenes, fatty acids, and phenolic compounds [16,17,18,19].

Although further pharmaco-chemical analyses are needed, here we hypothesize that the inhibition of COX activities may be more related to the presence of polyphenols. With respect to COX-1 inhibition, this may be associated with the presence of polysaccharides. Furthermore, the ethanolic extract of *B. x buttiana* is known to have the presence of flavonoids, as previously described [12,15,16,17,18,19]. The above confirms or explains the importance of polyphenols in COX-2 inhibition. Finally, it should be considered that, despite the notable inhibitory effects of most extracts on COX activity, none of them equaled the efficacy of the reference drug diclofenac.

While these compounds exhibit high biological activity in vitro, their effect is lower in vivo, possibly due to their ADME properties [17]. In silico analysis has been widely used to determine the physicochemical and pharmacokinetic properties of drug candidates during the preclinical research stage, helping to identify the appropriate route of administration [21,35]. In silico results showed that the physicochemical properties of the 16 compounds identified in the BxbREEs meet Lipinski’s RO5. The bioavailability radar plot showed that eight compounds (**C-1** to **C-6**, **C-10**, and **C-12**) displayed favorable drug-like properties and have good aqueous solubility. BxbREE-50% and BxbREE-80% were found to contain the compounds with the most favorable drug-like properties, HIA, and physicochemical profile, which may explain their highest anti-inflammatory activities in vitro. In contrast, **C-7** to **C-9**, **C-11**, and **C-13** to **C-16** exhibited slightly higher flexibility, low solubility, and high liposolubility, which can impact their bioavailability and absorption through the GI tract, suggesting that they cannot be administered orally. However, they can be administered systemically and/or could be incorporated into excipients to improve their solubility and bioavailability [17]. These results are consistent with those observed in the BOILED-Egg prediction. It is worth mentioning that the compounds with the highest probability of HIA and BBB were found in BxbREE-50% and BxbREE-80%, which are the extracts that show the highest anti-inflammatory activity in vitro and in vivo and confirms the drug-likeness of the compounds. Furthermore, it is important to note that the ability of the compounds to penetrate the BBB may be related to the effects on the central nervous system, such as the analgesic effect of *B. x buttiana* extracts reported in our previous studies [17,19]. Following oral administration and intestinal absorption, compounds enter the bloodstream and travel to the liver to be metabolized [36]. Due to the predictive drug-like properties of phenolic compounds (**C-3**, **C-5**, **C-6**, **C-10**, and **C12**), the abilities of these molecules to be substrates or inhibitors of CYP2C9, CYP42D6, CYP3A4, CYP1A2, and CYP2C19, which are some of the main CYP450 isoforms involved in drug metabolism, were evaluated. **C-12** was predicted as a CYP3A4 substrate, while all other compounds were not classified as substrates for the rest of the analyzed isoforms. On the other hand, it is relevant to recognize that CYP inhibition is one of the mechanisms involved in adverse reactions due to drug interactions [21,32,35,36]. **C-5** and **C-10** were identified as CYP1A2 inhibitors, and **C-12** as a CYP2A1 inhibitor. Overall, these results showed that four of the five phenolic compounds showed low CYP inhibitory promiscuity, except **C-12**. These results suggest that phenolic compounds from *B. x buttiana* are not metabolized by major phase I enzymes and have a low risk of drug–drug interactions, and it is important to remember that highly soluble compounds have a more limited distribution than liposoluble ones. Once absorbed, liposoluble compounds are distributed throughout the body, allowing them to reach tissues where they can interact with their molecular targets. This study used the SwissTargetPrediction web tool [25] to predict the top 15 human molecular targets with which the phenolic compounds might interact. The top three molecular targets most likely to interact with the five phenolic compounds, particularly **C-3** and **C-10**, were lyases, enzymes, and G protein-coupled receptors. Other molecular targets with which the phenolic compounds, particularly **C-5**, **C-6**, and **C-12**, might interact include CYP450 enzymes, nuclear receptors, and transcription factors. It is worth noting that, since enzymes are among the most likely molecular targets to interact, phenolic compounds may interact with enzymes involved in the inflammatory response, such as COX-1, COX-2, and PLA2. However, further experiments are required to corroborate this hypothesis. Phenolic compounds exhibit drug-likeness and favorable pharmacokinetic properties, making them strong candidates for future studies aimed at understanding the role of the compounds identified in BxbREE for their in vitro and in vivo anti-inflammatory effects.

## 4. Materials and Methods

### 4.1. Chemicals Reagents

Acetonitrile, bovine serum albumin (BSA), diclofenac sodium salt, 2-[(2,6-Dichlorophenyl)amino] benzeneacetic acid sodium salt (>98.5%), Ibuprofen α-Methyl-4-(isobutyl)phenylacetic acid, (±)-2-(4-Isobutylphenyl)propanoic acid (>98%), Tween-20, and ethanol were acquired from Sigma-Aldrich Chemical Co., Toluca, Estado de México, Mexico). The COX inhibitor detection assay kit (ovine/human), hG-IIA, pG-IB, and PLA2 from bovine pancreas were obtained from Cayman Chemical Co., (Ann Arbor, MI, USA).

### 4.2. Plant Material

Bracts were harvested in the municipality of Temixco, Morelos (18°47′40.70″ N and 99°11′49.27″ W, at an elevation of 1185 m). A specimen was stored and cataloged in the HUMO Herbarium, CIByC (UAEM), under the number 33,872, classified as *B. x buttiana* (var. Rose). The extraction process was performed as described in our previous study [12], and involved dehydrating the bracts at 25 °C, grinding them into powder, and mixing 2 g of this powder with a hydroalcoholic solution at 26 °C for 24 h. The mixture was processed in Whatman No. 1 filter paper (Whatman, London, UK), and the remains were extracted three more times. Three independent extractions were performed for each extract. The final residue was dehydrated using a rotary evaporator at 60 °C. The final yield of each BxbREE was calculated using the following formula:

$$\%yield=\left(\frac{Weight\;of\;extract\;material}{Weight\;of\;original\;plant\;material\;used}\right)\times 100$$

To assess the impact of the ethanol concentration on the in vitro anti-inflammatory activity and secondary metabolite profiles, the extracts were extracted using incrementing ethanol amounts (0%, 50%, 80%, and 100% *v*/*v*) and designated as BxbREE-0%, BxbREE-50%, BxbREE-80%, and BxbREE-100%.

### 4.3. In Vitro Anti-Inflammation Activity Determination

The in vitro anti-inflammatory activity of the BxbREEs was estimated using distinct assays, including heat-induced denaturation of trypsin and bovine serum albumin (BSA) denaturation, inhibition of pro-inflammatory enzyme activity (COX-1, COX-2, and PLA), and the stabilization of erythrocyte membrane assay.

#### 4.3.1. Denaturation Assay Using Heat-Induced Bovine Serum Albumin

The effects of BxbREE-0%, BxbREE-50%, BxbREE-80%, and BxbREE-100% on the heat-induced denaturation of bovine serum albumin (BSA) were estimated using the method described by Chandra et al., (2012) [8]. Briefly, each independent extraction of the reaction mixture was prepared with increasing quantities of the extracts: 100, 200, 300, 400, and 500 μg/mL, or diclofenac as a reference drug or phosphate-buffered saline (PBS) pH 6.4 as a control. All tubes that contained the mixtures were incubated at 37 °C for 20 min, and immediately after this time, the same mixtures were re-incubated at 70 °C for 5 min, and then after cooling, absorbance was estimated at 660 nm using a UV–visible spectrophotometer. Complete protein denaturation was identified as the control mixture without samples. The inhibition percentage of BSA denaturation was calculated using the following formula:

% inhibition of BSA denaturation = 100 − [(1 − AbsTs/AbsC) × 100].

where AbsC = absorbance of the control, and AbsTs = absorbance of the test sample.

#### 4.3.2. Proteinase Inhibitory Assay

The proteinase inhibitory activity of each ethanolic extract of *B. x buttiana* was assessed using the trypsin method [36]. Different concentrations of each extract obtained from 3 independent extractions (100, 200, 300, 400, and 500 μg/mL) were used. Diclofenac was used as a reference drug. To perform the assay, 1 mL of each test sample was mixed with 1 mL of solution, prepared with 0.06 mg of trypsin dissolved in Tris-HCl buffer (20 mM), and incubated for 15 min at 37 °C. After incubation, 1 mL of casein solution (0.8% *w*/*v*) was added, and the mixtures were further incubated at 37 °C. Following the 20 min of incubation, 2 mL of 70% perchloric acid were added to each sample. Next, the mixtures were centrifuged at 3000 rpm for 5 min. The supernatants carefully collected were used for the absorbance determination at 210 nm. The blank or standard solution was considered as the reaction mixture without the sample of each extract. The percentage of inhibition was calculated with the following formula:

% Inhibition of denaturation = [(1 − AbsTs/AbsControl) × 100]

where AbsC = absorbance of the control, and AbsTs = absorbance of the test sample.

### 4.4. Anti-Phospholipase Activity

The effects of BxbREE extracts (0%, 50%, 80%, and 100%) from 3 independent extractions on sPLA2 activity were quantified according to a previously reported protocol [17]. The reference drug was indomethacin (10 µg/mL). For this analysis, the group IIA isoforms of sPLA2, hG-IIA and pG-IB, were used. In brief, the substrate buffer was prepared with lecithin solubilized (3.5 mM) in NaCl (100 mM), sodium taurodeoxycholate (3 mM), CaCl_2_ (10 mM), and phenol red (0.055 mM) at pH 7.6. Next, 10 µL of each of the BxbREEs at the following concentrations, 100, 200, 300, 400, and 500 µg/mL, were mixed with 10 µL of PLA2-GIB and incubated at 25 °C for 20 min. Next, for each reaction mixture, 1 mL of PLA2 substrate was added, and the hydrolysis kinetics were supervised for 5 min by the absorbance measured at 558 nm. The inhibition percentage was determined by comparing the residual activity of the samples to the negative control (without BxbREEs). IC_50_ values were calculated using linear regression analysis.

### 4.5. Cyclooxygenase Inhibitory Activity of BxbREE

The effects of BxbREE-0%, BxbREE-50%, BxbREE-80%, and BxbREE-100% on COX-1 and COX-2 enzyme activities were assessed in vitro using a commercial COX kit (ovine/human) following the manufacturer’s instructions. The reference used was diclofenac (50 µg/mL). This kit measures the amount of PGF2a formed by the reduction of COX-derived PGH2 using SnCl_2_. The COX-2 activity was measured by use of 105 µL of reaction buffer and 10 µL of COX-1/2 enzymes. Like the COX-1/2 inhibitor plates, the effect of the extract was assessed by adding 20 µL of each BxbREE extract to each well in addition to the same ingredients. To inactivate COX-1 and COX-2 enzymes, the experimental samples were boiled for 3 min. In the initial reactions, 10 µL of arachidonic acid and 50 µL of 1 M HCl were added to each mixture. After the addition of SnCl_2_ (100 µL), the PGH_2_ formed is reduced, forming PGF_2_. The absorbance of each mixture was determined at 405 nm. The results were expressed as the percentage inhibition, and their IC_50_ values were determined by linear regression analysis.

### 4.6. Erythrocyte Membrane Stabilization Assay

The effects of the BxbREEs obtained with the concentrations of ethanol (-0%, -50%, -80%, and -100%) and independent extractions were evaluated using a modified method of stabilizing red blood cell membranes in two steps through lysing, as previously described [37,38]. Step 1, for obtaining red blood cell stock, involved human whole blood samples (5 mL) collected through venipuncture placed into tubes containing the anticoagulant ethylenediaminetetraacetic acid (EDTA). The tubes with blood samples were centrifuged at 3000 rpm for 10 min at 25 °C. After this time, the supernatants were collected and discarded. The resulting cell concentrates were washed three times with a similar volume of isosaline solution (0.85% *w*/*v* NaCl) until a transparent supernatant was achieved. Next, the stock red blood cell suspension was maintained in isosaline solution (10% *v*/*v*). For step 2, the membrane stabilizing activity assay, the reaction mixtures were prepared in tubes as follows: each reaction mixture constituted the addition of 1 mL of PBS (pH 7.4, 0.15 mol/L), 2 mL of hyposaline solution prepared with (0.36% *w*/*v* NaCl), 0.5 mL of a 10% (*v*/*v*) human erythrocyte suspension, and 0.5 mL of increasing concentrations of each BxbREE or the reference drug (diclofenac sodium). For the control mixture, the hyposaline solution was replaced with distilled water to induce 100% hemolysis. All tubes, containing reaction mixtures, test samples, and/or the control, were incubated in a water bath at 56 °C for 30 min, and then centrifuged at 5000 rpm at room temperature. At the end of 5 min, the absorbance (Abs) of the supernatant at 540 nm was measured. The percentage of membrane stabilization was calculated using the following formula:

% of membrane stabilization = (AbsBxbREE/AbsControl − 1) × 100

### 4.7. In Silico Evaluation of Pharmacokinetics Properties, Physicochemical Profile, and Drug-Likeness for Compounds in BxbREE

The compounds identified in each BxbREE were analyzed using the SwissADME web tool [21]. This software allows us to calculate the ADME (Absorption, Distribution, Metabolism, and Excretion) properties and checked the Lipinski’s rule of five (RO5), drug-likeness, and oral bioavailability. With this tool, the molecular descriptions that were estimated were as follows: the molecular weight (MW), the number of hydrogen bond donors or acceptors, the partition coefficient between n-octanol and water (Log P), and the compliance with the criteria of Lipinski’s rule of five (RO5) [22]. For absorption, the water solubility and bioavailability index were calculated. Additionally, the synthetic accessibility was calculated. To assess the bioavailability and drug-likeness, parameters such as hydrophobicity, electron distribution, hydrogen bond characteristics, molecular size, and flexibility were estimated. The results of this assessment were presented in a radar plot. Moreover, we conducted the BOILED-Egg test to predict passive human gastrointestinal absorption (HIA), blood–brain barrier (BBB) penetration, and P-glycoprotein efflux of the compounds **C1** to **C16** [39]. The “BOILED-Egg” is a graphical representation of HIA as a function of the position of the small molecule on the WLOGP vs. TPSA plot. In this graphical representation, the white region indicates high HIA, while the yellow area indicates the high probability of penetration of the BBB. In addition, dots are colored green if the compound is actively effluxed by P-glycoprotein (P-gp^+^) and red if it is predicted not to be a non-substrate of P-glycoprotein (P-gp^−^). In the same way, due to the drug-like properties of the phenolic compounds (**C-3, C-5, C-6, C-10**, and **C-12**), we predicted their potential to act as inhibitors or substrates of the main cytochrome enzymes involved in phase I of drug metabolism. Additionally, to expand the knowledge about the pharmacodynamics, the ligand-based target prediction for humans was done using the web tool SwissTargetPrediction, a web-based tool, available on-line since 2014 (www.swisstargetprediction.ch) [25].

### 4.8. Presentation of Results and Statistical Analysis

Data are presented as mean ± standard deviation (SD). Half inhibitory concentration (IC_50_) values for COX and phospholipases were determined using linear regression. Statistical significance was determined using one-way ANOVA followed by a post hoc Dunnett’s test. *p* values < 0.05 were considered statistically significant. The IC50 calculation and statistical analysis were performed using GraphPad Prism V8 (GraphPad Software, Inc., San Diego, CA, USA).

## 5. Conclusions

Our results showed that increasing ethanol quantities in the bracts of BxbREE preparation induce a decrease in the final efficiency of the extracts. Nonetheless, the increase in ethanol quantity significantly increments the in vitro anti-inflammatory actions of BxbREE-50% and BxbREE-80% when compared to BxBREE-0% and BxbREE-100%. These extracts were competent to inhibit BSA denaturation, trypsin denaturation, and PLA2 and COX activities, while also protecting erythrocyte hemolysis. These combined results rescue the use of *B. x buttiana* in traditional Mexican medicine. Furthermore, the pharmacological activities of this plant can be attributed to the different metabolites identified in ethanolic extracts. In the in silico analysis, it showed that many of the compounds identified in the BxbREEs met the Lipinski RO5 criteria, displayed favorable ADME properties, and showed adequate bioavailability for oral administration. Notably, the compounds with the best drug-like properties were phenolic, primarily found in BxbREE-50% and BxbREE-80%, suggesting that they play an essential role in the anti-inflammatory effect of *B. x buttiana*. However, further in vitro and in vivo studies are still needed to extend our knowledge about the properties, such as the pharmacokinetics and pharmacodynamics, of the bioactive compounds from *B. x buttiana*, including protein–ligand interaction assays, to assist the progress of the development of new therapeutic agents.

## Figures and Tables

**Table 1:** Ethanol quantity effect on the recovery of BxbREEs.

Ethanol Concentration in the Extract	Recovery (%)
0%	30.31 ± 0.69 *
50%	29.28 ± 0.72 *
80%	19.49 ± 0.51 *
100%	7.82 ± 0.18

* (*p* < 0.001) vs. the BxBREE-0% by one-way ANOVA followed by the Dunnett test.

**Table 2:** Phase I metabolism of phenolic compounds present in BxbREE.

Clave	C-3	C-5	C-6	C-10	C-12
Chemical name	2-Propenoic acid, 3-(2-hydroxyphenyl)-, (E)-	Benzofuran, 2,3-dihydro-	2-Methoxy-4-vinylphenol	Ethanone, 1-(2-hydroxy-5-methylphenyl)-	Naphthalene, 3,4-dihydro-1,8-bis(trimethylsilyloxy)-
Identified in:	BxbREE-0%, -50%, -80%, -100%	BxbREE-50%	BxbREE-50%, -80%	BxbREE-80%	BxbREE-100%
CYP isoform					
CYP450 2C9 Substrate/ Inhibitor	Non-substrate/ non-inhibitor	Non-substrate/ non-inhibitor	Non-substrate/ non-inhibitor	Non-substrate/ non-inhibitor	Non-substrate/ non-inhibitor
CYP450 2D6 Substrate/Inhibitor	Non-substrate/non-inhibitor	Non-substrate/ non-inhibitor	Non-substrate/non-inhibitor	Non-substrate/ non-inhibitor	Non-substrate/ non-inhibitor
CYP450 3A4 Substrate/Inhibitor	Non-substrate/ non-inhibitor	Non-substrate/ non-inhibitor	Non-substrate/non-inhibitor	Non-substrate/ non-inhibitor	Substrate/ non-inhibitor
CYP450 1A2 Inhibitor	Non-inhibitor	Inhibitor	Non-inhibitor	Inhibitor	Inhibitor
CYP450 2C19 Inhibitor	Non-inhibitor	Inhibitor	Non-inhibitor	Non-inhibitor	Inhibitor
CYP Inhibitory Promiscuity	Low CYP inhibitory promiscuity	Low CYP inhibitory promiscuity	Low CYP inhibitory promiscuity	Low CYP inhibitory promiscuity	High CYP inhibitory promiscuity

## Data Availability

The original contributions presented in the study are included in the article and supplementary material, further inquiries can be directed to the corresponding author.

## Supplementary Materials

The following supporting information can be downloaded at: https://www.mdpi.com/article/10.3390/ph18060821/s1. Table S1: Physicochemical profile of the bioactive compounds found in BxbREEs.

## Abbreviations

The following abbreviations are used in this manuscript:

BSA	Bovine serum albumin
BxbREE	*B. x buttiana* (var. Rose) ethanolic extracts
**C-1**	2,5-Dimethyl-4-hydroxy-3-(2H)-Furanone
**C-2**	4H-pyran-4-one, 2,3-dihydro-3,5-dihydroxy-6-methyl
**C-3**	2-Propenoic acid, 3-(2-hydroxyphenyl)-, (E)-
**C-4**	3-O-Methyl-d-glucose
**C-5**	Benzofuran, 2,3-dihydro
**C-6**	2-Methoxy-4-vinylphenol
**C-7**	2-Methoxy-4-vinylphenol
**C-8**	Hexadecanoic acid, ethyl ester
**C-9**	9,12-Octadecadienoic acid, ethyl ester
**C-10**	Ethanone, 1-(2-hydroxy-5-methylphenyl)-
**C-11**	*n*-Hexadecanoic acid
**C-12**	Naphthalene, 3,4-dihydro-1,8-bis(trimethylsilyloxy)-
**C-13**	9,12-Octadecadienoic acid (Z,Z)-
**C-14**	9-Octadecenoic acid
**C-15**	Octadecanoic acid
**C-16**	Stigmasta-5,22-dien-3-ol
COX-1	Cyclooxygenase-1
COX-2	Cyclooxygenase-2
CYP450	Cytochrome P450
hG-IIA	Human group IIA secreted phospholipase A2
pG-IB	Secretory phospholipase A2 group IB
PLA2	Phospholipase A2
