# Supplementary material for: Parallel In Vitro and In Silico Studies of the Anti-Inflammatory Activity of Bioactive Compounds Found in Different Ethanolic Extracts of Bracts from B. x buttiana (var. Rose): A Comparative Analysis
Source: Pharmaceuticals (Basel). 2025 May 30;18(6):821. doi: 10.3390/ph18060821 (PMC12196137; doi:10.3390/ph18060821)
Supplement: Supplementary file 1 [file pharmaceuticals-18-00821-s001.zip › pharmaceuticals-3633333-supplementary.pdf]

Supplementary Table S1. Physicochemical profile of the bioactive compounds found in BxbREEs.

| Clave | MW | nHBD | nHBA | cLogP | Meet Lipinski Ro5 Criteria | | Water Solubility LogS | | Bioavailability Score | Synthetic accessibility |
|---|---|---|---|---|---|---|---|---|---|---|
| | <500 | <5 | <10 | <5 | $N_{vio}$ <1 | Yes/No | (ESOL) | (Ali) | | |
| C-1 | 128.13 | 1 | 3 | 0.56 | 0 | Yes | -1.06; **VS** | -1.23; **VS** | 0.85 | 3.26 |
| C-2 | 144.13 | 2 | 4 | -0.22 | 0 | Yes | -0.50; **VS** | -0.57, **VS** | 0.85 | 3.60 |
| C-3 | 164.16 | 2 | 3 | 1.40 | 0 | Yes | -2.37; **S** | -2.87, **S** | 0.85 | 1.85 |
| C-4 | 194.18 | 4 | 6 | -1.70 | 0 | Yes | 1.17; **HS** | 1.20; **HS** | 0.55 | 3.52 |
| C-5 | 120.15 | 0 | 1 | 2 | 0 | Yes | -2.43; **S** | -1.97; **VS** | 0.55 | 1.49 |
| C-6 | 150.17 | 1 | 2 | 2.14 | 0 | Yes | -2.81; **S** | -3.09; **S** | 0.55 | 1.45 |
| C-7 | 270.45 | 0 | 2 | 5.54 | 1 | Yes | -5.18; **MS** | -7.76; **PS** | 0.55 | 2.53 |
| C-8 | 284.48 | 0 | 2 | 5.90 | 1 | Yes | -5.51; **MS** | -8.28; **PS** | 0.55 | 2.80 |
| C-9 | 308.50 | 0 | 2 | 6.09 | 1 | Yes | -5.32; **MS** | -7.72; **PS** | 0.55 | 3.34 |
| C-10 | 150.17 | 1 | 1 | 1.94 | 0 | Yes | -2.55; **S** | -2.70; **S** | 0.55 | 1.00 |
| C-11 | 256.42 | 1 | 2 | 5.20 | 1 | Yes | -5.02; **MS** | -7.77; **PS** | 0.85 | 2.31 |
| C-12 | 306.55 | 0 | 2 | 3.78 | 0 | Yes | -5.17; **MS** | -5.66; **MS** | 0.55 | 3.79 |
| C-13 | 280.45 | 1 | 2 | 5.88 | 1 | ND | ND | ND | ND | ND |
| C-14 | 282.46 | 1 | 2 | 5.65 | 1 | Yes | -5.41; **MS** | -8.26; **PS** | 0.85 | 3.07 |
| C-15 | 284.48 | 1 | 2 | 5.93 | 1 | Yes | -5.73; **MS** | -8.87; **PS** | 0.85 | 2.54 |
| C-16 | 412.69 | 1 | 1 | 6.98 | 1 | Yes | -7.46; **PS** | -8.86; **PS** | 0.55 | 6.21 |

MW: molecular weight; nHBD: number of hydrogen-bond donors; nHBA: number of hydrogen bond acceptors; cLog P: logarithm of partition coefficient of the compound between n-octanol and water; NVio: number of RO5 violated; TPSA: topological polar surface area; nRotB: Num. rotatable bonds. Water solubility Log S (S. soluble, VS Very soluble, HS Highly soluble, MS Moderate soluble, PS Poor soluble). C-1: 2,5-Dimethyl-4-hydroxy-3-(2H)-Furanone; C-2: 4H-pyran-4-one, 2,3-dihydro-3,5-dihydroxy-6-methyl; C-3: 2-Propenoic acid, 3-(2-hydroxyphenyl)-, (E)-; C-4: 3-O-Methyl-d-glucose; C-5: Benzofuran, 2,3-dihydro-; C-6: 2-Methoxy-4-vinylphenol; C-7: Hexadecanoic acid, methyl ester; C-8: Hexadecanoic acid, ethyl ester; C-9: 9,12-Octadecadienoic acid, ethyl ester; C-10: Ethanone, 1-(2-hydroxy-5-methylphenyl)-; C-11: *n*-Hexadecanoic acid; C-12: Naphthalene, 3,4-dihydro-1,8-bis(trimethylsilyloxy)-; C-13: 9,12-Octadecadienoic acid (Z,Z)-; C-14: 9-Octadecenoic acid (E)-; C-15: Octadecanoic acid; C-16: Stigmasta-5,22-dien-3-ol.
